# Distal Radius Fracture Management: Surgeon Factors Markedly Influence Decision Making

**DOI:** 10.5435/JAAOSGlobal-D-23-00002

**Published:** 2023-03-02

**Authors:** Alex Doermann, Deven K. Gupta, David J. Wright, Babar Shafiq, Jacques Hacquebord, Gregory Rafijah, Philip K. Lim, Ranjan Gupta

**Affiliations:** From the Department of Orthopaedic Surgery, University of California, Irvine, Irvine, CA (Dr. Doermann, D.K. Gupta, Dr. Wright, Dr. Rafijah, Dr. Lim, and Dr. R. Gupta); the Department of Orthopaedic Surgery, Johns Hopkins University, Baltimore, MD (Dr. Shafiq); and the Department of Orthopaedic Surgery, New York University Langone Health, New York, NY (Dr. Hacquebord).

## Abstract

It is our hypothesis that physician-specific variables affect the management of distal radius (DR) fractures in addition to patient-specific factors.

**Methods:**

A prospective cohort study was conducted evaluating treatment differences between Certificate of Additional Qualification hand surgeons (CAQh) and board-certified orthopaedic surgeons who treat patients at level 1 or level 2 trauma centers (non-CAQh). After institutional review board approval, 30 DR fractures were selected and classified (15 AO/OTA type A and B and 15 AO/OTA type C) to create a standardized patient data set. The patient-specific demographics and surgeon's information regarding the volume of DR fractures treated per year, practice setting, and years posttraining were obtained. Statistical analysis was done using chi-square analysis with a postanalysis regression model.

**Results:**

A notable difference was observed between CAQh and non-CAQh surgeons. Surgeons in practice longer than 10 years or who treat >100 DR fractures/year were more likely to choose surgical intervention and obtain a preoperative CT scan. The two most influential factors in decision making were the patients' age and medical comorbidities, with physician-specific factors being the third most influential in medical decision making.

**Discussion:**

Physician-specific variables have a notable effect on decision making and are critical for the development of consistent treatment algorithms for DR fractures.

Across surgical disciplines, it is the belief and expectation that a patient's injury, value-based preferences unclouded by common misconceptions, and health status should determine their ideal treatment course. There have been notable efforts made to reduce physician variability by establishing classification systems and subsequent treatment algorithms.^1^ Although these efforts have been successful for many medical conditions, there remains broad variability in the surgical treatment of fractures of the distal end of the radius. Distal radius (DR) fractures (DRFs) are one of the most common orthopaedic fractures, accounting for more than 15% of all bony injuries with an annual incidence of >600,000. They have a bimodal distribution, affecting younger patients typically with higher energy injuries and elderly patients (age >65 years of age) typically with lower energy mechanisms. Appropriate management of DRFs necessitates detailed fracture assessment, diagnosis, treatment, and evaluation of outcomes. Given their universality, they are treated by a myriad of surgeons across subspecialty designations, ranging from academic hand surgeons to rural general orthopaedic surgeons.

Previous efforts have been made to guide treatment based on fracture findings and patient demographic information. However, DRFs are notoriously difficult to classify, with poor intraobserver and interobserver reliability among all specialties, including among certificate of additional qualification (CAQ) hand surgeons (CAQh).^2-6^ The Lafontaine criteria have been used to determine whether surgical management should be recommended.^7^ There have also been hospital-based guidelines to create a treatment algorithm and Clinical Practice Guidelines of the American Academy of Orthopaedic Surgeons.^8,9^ In contrast to evaluating patient-specific factors, there have been no previous evaluations of the potential influence of physician demographic data for the treatment of DRFs. Although no two surgeons are identical, it is assumed that treatment decision making is influenced by one's subspecialty training, hospital setting, surgical experience, and the volume of fractures treated per year. Before developing the objective consistent guidelines for the treatment of an injury treated by a diverse group of orthopaedic surgeons, it is important to establish if there is an inherent bias for particular types of treatments based on these surgeon-specific factors.

## Methods

### Study Design

A prospective cohort study was conducted evaluating differences in treatment between CAQ hand surgeons and board-certified orthopaedic surgeons who take call at a level 1 or level 2 trauma center (non-CAQ surgeons). The two cohorts included 25 CAQ hand surgeons and 25 non-CAQ surgeons, with a total N of 50. After institutional review board approval, a retrospective chart review was done for any patient aged 18 years or older who sustained a DRF between January 1, 2018, and January 1, 2020. All subjects had plain radiographs with both prereduction and postreduction images, and a CT scan was required for at least 15 of the 30 fractures. Subjects were excluded if they had multiple concurrent injuries to the ipsilateral ulnar shaft or distal ulna. After a review of the >75 fractures that fit these criteria during this period, 30 DRFs were selected based on their age and fracture AO/OTA classification (15 AO/OTA type A and B and 15 AO/OTA type C) to create a standardized patient data set. Fifteen AO/OTA type C were selected, given their propensity to be an injury that would require surgical intervention. The classification was done by three CAQ hand surgeons independently. Any discrepancies between fracture classifications were discussed and agreed upon before the final selection of fractures.

A deidentified presentation was used to sequentially display radiographic images followed by patient-specific demographics. The surgeons being evaluated were provided a treatment survey document (Appendix A, http://links.lww.com/JG9/A272) before testing. The survey included nine questions that were sequentially asked for each of the 30 DRFs. All testing was done remotely using the Zoom platform. The data points as presented during analysis included (1) prereduction and postreduction radiographic images, (2) CT radiographs (15 of 30 fractures), (3) patient's age, (4) notable medical comorbidities, (5) patient's manual laborer status, and (6) associated polytrauma. The surgeon's fracture management was inquired after each of the above data points was consecutively provided based on the treatment survey. Treatment options included both closed (splint in situ and closed reduction and casting) and open management options (closed reduction and pinning ± external fixator, open reduction and internal fixation with fragment specific or volar locking plate, and dorsal spanning plate ± adjuvant fixation). After the survey was completed, demographic information about the surgeon was ascertained, including number of DRF treated per year, number of years postfellowship training, and their current practice setting.

### Statistical Methods

Statistical analysis was done using chi-square analysis comparing treatment selections based on their fellowship training status (CAQ and non CAQ hand surgeons), years of experience postfellowship, frequency of treatment of DRFs, and their practice setting. Biostatistics, epidemiology, and research design statistics were used to perform a regression analysis.

## Results

Of the fracture subjects, 40% were male and 60% were female. The mean age of the patients was 53.5 ± 21.1 years, spanning a bimodal distribution. Fifty percent of the patients noted that their occupation required manual labor, 70% were marked with having a comorbidity, and 33% had an associated polytrauma. Regarding the physician cohorts, this study included 50 physicians, with an average of 13.3 ± 11.1 years of practicing medicine. Furthermore, regarding physician characteristics, the mean amount of DRFs attended to each physician per year was 100.1 ± 120.3 DRFs. Nine were hospital-employed, and the other 41 were not.

**Figure 1:**
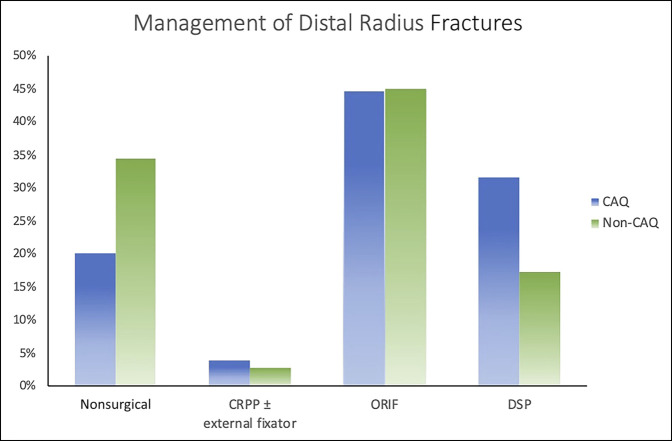
Graph showing the management of distal radius fractures. CAQ = Certificate of Additional Qualification, CRPP = Closed Reduction and Percutaneous Pinning, ORIF = Open Reduction and Internal Fixation, DSP = Dorsal Spanning Plate

Using a chi-square analysis, we found that there was a significant difference between CAQh and non-CAQh surgeons regarding the management of DRFs (*P* < 0.001). When deciding on DR management, CAQh surgeons are more likely to perform surgical fixation than non-CAQ surgeons (90% versus 66%, *P* < 0.001) and are almost twice as likely to use a dorsal spanning plate (24% versus 14%, *P* < 0.001). Figure 1 displays the management of CAQh physicians compared with non-CAQh physicians of DRFs. Hospital-employed surgeons were more likely to change their management based on the patients' age (22% versus 14%, *P* = 0.001). Nonhospital-employed surgeons also tended to be more likely to apply a dorsal spanning plate (20% versus 14%, *P* = 0.07). Surgeons who independently have either been in practice longer than 10 years or who treat >100 DR fractures per year were more likely to choose surgical intervention and obtain a CT scan in their preoperative planning than surgeons who had been in practice less than 10 years or treat less than 100 DRF per year (54% and 50% versus 39% and 43%, *P* < 0.001 and *P* = 0.042, respectively). After performing a subgroup analysis of the non-CAQh surgeons, non-CAQ traumatologists (15/25) were less likely to opt for percutaneous fixation with or without external fixation than nontraumatologists and more likely to opt for dorsal spanning plates with or without adjuvant fixation (12.1% and 0.6% versus 4.2% and 0.1%, *P* = 0.007 and *P* < 0.001, respectively).

**Figure 2:**
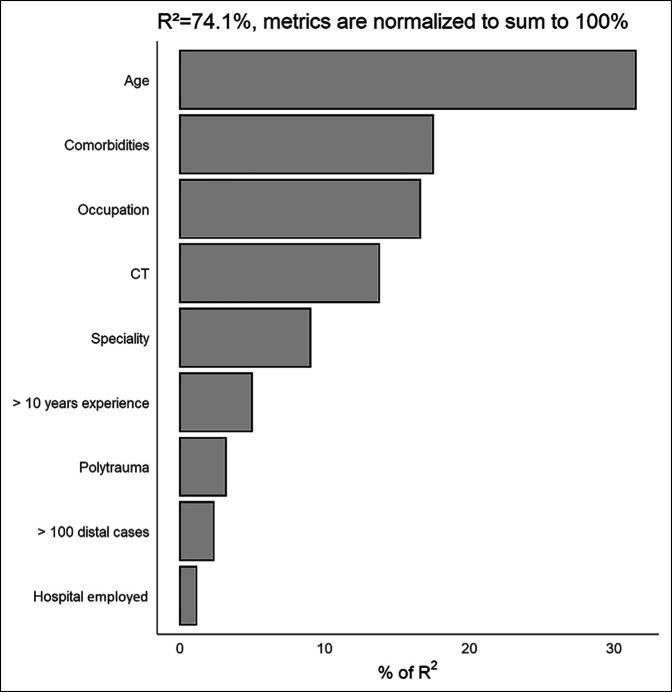
Graph showing the full model with all patient and physician factors (17b).

**Figure 3:**
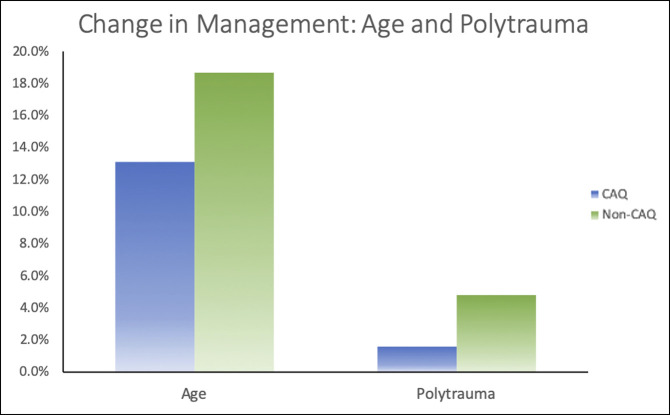
Graph showing the change in management: age and polytrauma. CAQ = Certificate of Additional Qualification

In our regression analysis, the two most influential factors in decision making were the patients' age and medical comorbidities (32% and 18%, respectively); however, physician-specific factors are the third most influential in medical decision making, accounting for 17% of the changes in treatment management (Table 1). Of the remaining patient characteristics, occupation accounted for 17% in decision making, the presence of a CT accounted for 14%, and polytrauma accounted for 3%. Of the physician-specific factors, a surgeon's CAQh qualification (9%) was the most significant and hospital employed was the least significant (1%). Figure 2 displays the decision-making factors compared with each other. Figure 3 displays the differences in management between CAQh and non-CAQh physicians based on patient age and polytrauma.

**Table 1:** Relative Importance Analysis

Factor	Relative Importance Weight (%)
Patient characteristics	
Age	32
Occupation	17
Comorbidities	18
CT	14
Polytrauma	3
Physician characteristics	
Specialty	9
Hospital employed	1
>10 years of experience	5
>100 distal cases	2

## Discussion

This study elucidated many interesting results that affected the treatment of DRFs. DRFs are highly prevalent, accounting for more than 15% of all bony injuries with an annual incidence of >600,000.^2^ Despite advancements in surgical technique and philosophy, discrepancies still occur among physicians in DR treatment preferences. As such, DR management is important to standardize across orthopaedic physicians, regardless of the level of training. Our study attempts to examine treatment preferences in DRF fixation based on physician-specific and patient-specific factors.

Our study confirms previous notions that a patient's age and medical comorbidities are the most important factors when deciding on surgical treatment. In a 2009 meta-analysis of 107,190 patients by Fanuele et al, physicians were more likely to choose nonsurgical treatment for DRF for older patients (age > 85 years) with an odds ratio of 2.9. In addition, they found medical comorbidities to have opposing effects on the likelihood of having surgery with an increased amount of comorbidities increasing the likelihood for a physician to proceed with open reduction and internal fixation (odds ratio, 1.9) and decreasing the likelihood for a physician to choose a percutaneous fixation (odds ratio, 0.19).^10^ Our study furthered this understanding by adding physician factors as a separate category to be analyzed, allowing all three factors to be weighed comparatively regarding their effects on the decision making associated with DRF fixation. Physician factors account for 17% of management decisions during surgery. These factors were the third most influential in medical decision making, following the two most influential factors: patients' age and medical comorbidities (32% and 18%, respectively). Although previous studies have analyzed DRFs with an emphasis on physician-specific factors, our study is the most holistic in identifying the extent to which these factors contribute to the decision making.^11,12^ In addition, in comparison with previous studies, ours presented the most comprehensive range of surgical and nonsurgical management techniques for physicians to decide between.^11,12^ Although it is not surprising that patient age and medical comorbidities play a large role in DR management, the high percentage of decision making attributed to physician-specific factors illuminates an element of subjectivity in the current management of DRFs.

Although patient-specific factors do alter the management, this study also details that a surgeon's CAQh qualification was the most notable physician-specific factor that affected DRF treatment preference. Although the effect of member status of the American Society for Surgery of the Hand has been recognized particularly in the management of geriatric DRFs, the effect of CAQh qualification has not previously been documented.^11,13^ Although the previous study found that the American Society for Surgery of the Hand member status accounted for 12% of the variance in DRF management, we find that CAQh qualification status accounts for 9% and that this applies to patients of all age groups with DRFs.

CAQh and more experienced (>10 years in clinical practice and >100 DRFs treated per year) surgeons are more likely to use a dorsal spanning plate. However, this device is underutilized by non-CAQ and hospital-employed surgeons for the fixation of DRFs. Yet our subgroup analysis showed that non-CAQ traumatologists trend with CAQh toward the use of dorsal spanning plate fixation. In an evaluation of the evolution of DR management over time, Koval et al^14^ found that younger orthopaedic surgeons opt for open treatment, especially volar locked plating, as opposed to percutaneous fixation of distal radial fractures. Although the study does not conclude about the explanation to this recent trend, the increased preference by younger physicians to volar locked plating could explain the difference caused by the preference of more experienced surgeons to use a dorsal spanning plate instead of other alternatives, such as volar locked plating. Other studies find that volar plating is usually preferred because of the assumed lower complication frequency.^15,16^ Hannemann et al^17^ rebutted this, noting that these current assumptions do not account for newer generation low-profile dorsal plates, which have statistically similar complication rates as volar plates. They conclude that the decision for fracture fixation should be based on fracture type and the surgeon's experience with the specific approach and plate types. Thus, we deduce that younger surgeons with less experience and/or training are more likely to opt for using a volar plate than a dorsal plate as they have more experience with the former. Moreover, as these groups have less experience with DRF fixation decision making, it is probable that they are more likely to choose the option with the assumed lower complication frequency, which may explain the results found in our study.

Surgeons with more experience are more likely to obtain a CT scan in their preoperative planning and choose surgical intervention as their definitive management. It has been found that experienced surgeons can predict the usefulness of CT scans for intra-articular displaced DRFs to decide on treatment, but for preoperative planning, the usefulness of CT scans is much harder to predict.^18^ Although our study did not address potential reasons that address the causation between surgical experience with the likeliness of obtaining a CT and choosing an surgical intervention, several reasons may explain this effect. Experienced surgeons likely have greater exposure to evidence regarding the relative effectiveness of treatment strategies for DRFs. They also may have increased exposure to new fixation or surgical techniques because of differences in marketing strategies by biomedical companies toward subspecialty groups and experienced surgeons. Future studies are necessary to accurately understand this relationship.

This study had a few limitations. As we only researched the correlation between CAQ hand qualification status and DRF management, we cannot conclude to what extent current algorithms to standardize DRFs have affected modern surgeons. However, since our study still finds differences in treatment preferences between surgeons of different experience and training levels, our results elucidate that either of these methods is being underutilized or there are structural problems with them. Additional experiments could be done comparing algorithmic management of DRFs with subcategories for physician subjects based on levels of training. Moreover, our study evaluated physician experience discretely, with surgeons classified by practicing either shorter or longer than 10 years or treating either more or less than 100 DRFs per year. Future studies could analyze these data using continuous measurements so as to further represent how surgical experience affects DRF management.

DRF management is an important issue in orthopaedics that has markedly evolved over the past few decades. This study suggests that physician-specific variables have a notable effect on decision making and are critical to account for in the development of consistent objective treatment algorithms for DRFs.
